# Enhanced Fine-Form Perception Does Not Contribute to Gestalt Face Perception in Autism Spectrum Disorder

**DOI:** 10.1371/journal.pone.0170239

**Published:** 2017-02-01

**Authors:** Takao Yamasaki, Toshihiko Maekawa, Yuka Miyanaga, Kenji Takahashi, Naomi Takamiya, Katsuya Ogata, Shozo Tobimatsu

**Affiliations:** 1 Department of Clinical Neurophysiology, Neurological Institute, Graduate School of Medical Sciences, Kyushu University, Fukuoka, Japan; 2 Department of Neurology, Minkodo Minohara Hospital, Fukuoka, Japan; 3 Department of Physical Therapy, Faculty of Health and Welfare, Prefectural University of Hiroshima, Hiroshima, Japan; University of Toyama, JAPAN

## Abstract

Individuals with autism spectrum disorder (ASD) show superior performance in processing fine detail, but often exhibit impaired gestalt face perception. The ventral visual stream from the primary visual cortex (V1) to the fusiform gyrus (V4) plays an important role in form (including faces) and color perception. The aim of this study was to investigate how the ventral stream is functionally altered in ASD. Visual evoked potentials were recorded in high-functioning ASD adults (n = 14) and typically developing (TD) adults (n = 14). We used three types of visual stimuli as follows: isoluminant chromatic (red/green, RG) gratings, high-contrast achromatic (black/white, BW) gratings with high spatial frequency (HSF, 5.3 cycles/degree), and face (neutral, happy, and angry faces) stimuli. Compared with TD controls, ASD adults exhibited longer N1 latency for RG, shorter N1 latency for BW, and shorter P1 latency, but prolonged N170 latency, for face stimuli. Moreover, a greater difference in latency between P1 and N170, or between N1 for BW and N170 (i.e., the prolongation of cortico-cortical conduction time between V1 and V4) was observed in ASD adults. These findings indicate that ASD adults have enhanced fine-form (local HSF) processing, but impaired color processing at V1. In addition, they exhibit impaired gestalt face processing due to deficits in integration of multiple local HSF facial information at V4. Thus, altered ventral stream function may contribute to abnormal social processing in ASD.

## Introduction

Autism spectrum disorder (ASD) is a neurodevelopmental condition characterized by alterations in social communication and interaction, co-occurring with restricted, repetitive patterns of behavior, interests or activities [1]. In addition to these core deficits, individuals with ASD display atypical perceptual processing (i.e., enhanced simple and lower-level processing, but impaired complex and higher-level processing) during visual [2] and auditory behavioral tasks [3]. In the visual domain, ASD is associated with superior performance in detailed form (local structure) perception, but impairment of face perception [2,4,5]. For example, individuals with ASD are often faster and better at identifying target stimuli in visual search tasks than typically developing (TD) individuals [6], segmenting and constructing patterns in the Block Design task [7], and locating shapes in the Embedded Figures Test [8]. In contrast, several aspects of face processing are impaired in ASD, including anomalies in memory for facial identity [9], recognition of facial expressions of emotion [10] and gaze processing [10]. Interestingly, these atypical visual perceptions may be primary, or at least contributory, to abnormalities in social processing in ASD [4].

Fine-form perception is mainly processed in the ventral stream, which begins with the primary visual cortex (V1) via the parvocellular system in the lateral geniculate nucleus (LGN), and projects to the fusiform gyrus (V4) and the inferior temporal (IT) cortex. In contrast, motion perception is processed in the dorsal stream beginning with V1 via the magnocellular system in the LGN, and projecting to the V5/middle temporal (MT) area and posterior parietal cortex, thus giving rise to parallel visual information processing [11,12]. The ventral stream is divided into color (blob) and form (inter-blob) pathways. The former is dedicated to color perception for color constancy, while the latter contributes to form perception with a preference for the high spatial frequency (HSF) component of the visual world [11]. The form pathway is characterized by hierarchical architecture; neurons in V1 code for relatively simple features such as local contours, whereas neurons in V4 and IT fire in response to whole complex forms (e.g., words and faces) [13]. These distinct features derive from the specific physiological characteristics of the ventral and dorsal streams. The ventral stream is characterized by color sensitivity, high spatial resolution, low contrast sensitivity and low temporal resolution. In contrast, the dorsal stream exhibits opposite natures: color insensitivity, low spatial resolution, high contrast sensitivity and high temporal resolution [11,12]. Based on parallel visual processing, enhanced V1 function with impaired V4 function of the form pathway within the ventral stream could explain unique behaviors such as superior fine-form perception with inferior face processing in ASD.

Visual evoked potentials (VEPs) are visually evoked electrophysiological signals that can be studied non-invasively, and provide objective information concerning the function of the visual system [12]. By manipulating the visual stimulus parameters of VEPs, we can separately explore the function of each visual pathway at different hierarchical levels based on the distinct physiological characteristics of each visual pathway [12,14]. Regarding the ventral stream, the isoluminant chromatic (red/green, RG) and achromatic (black/white, BW) gratings with HSF can selectively stimulate color and form pathways at the V1 level, respectively [15]. The occipital N1 is a major component in both stimuli [15]. Face stimuli are useful for sequentially evaluating the form pathway from V1 to V4. The right-lateralized occipital P1 (V1 origin) and occipitotemporal N170 (V4 origin) are elicited as major components [16–21]. In contrast, BW gratings with high temporal frequency [22] and coherent motion are suitable for evaluating the dorsal stream function at the lower (V1) and higher (V5/MT and parietal cortex) levels, respectively [20,23].

We recently evaluated the function of each visual stream at different levels in individuals with ASD [14,22–24]. Specifically, we found a prolonged N1 latency for RG gratings and preserved VEPs for BW gratings with high temporal frequency. These findings implied impairment of color pathway function, but preserved dorsal stream function at the lower (V1) level [22]. We also demonstrated the prolongation of VEP latencies for coherent motion, which indicated impairment of higher-level dorsal stream (V5/MT and parietal cortex) function [23]. We are yet to study face perception systematically, however, some VEP studies reported prolonged N170 latency [25–27] while others did not [28–30]. Thus, the neural basis of face processing at V4 may be anomalous in ASD. Overall, it has never been systematically investigated how form and color pathways within the ventral stream at lower (V1) and higher (V4) levels are functionally altered in ASD.

Therefore, in the present study, we focused on the functions of form and color pathways in individuals who are TD and those with ASD using VEPs with isoluminant RG gratings, BW gratings with HSF and face stimuli. Based on the results of previous behavioral [2,4] and VEP studies [14,22–27], we hypothesized that ASD adults exhibit enhanced function of the form pathway with impaired function of the color pathway at V1 and impaired gestalt face processing at V4.

## Materials and Methods

### Participants

Fourteen individuals with ASD (five females; 29.9 ± 7.1 years) and 14 control TD (five females; 28.6 ± 10.0 years), matched for age, sex, and intelligence quotient (IQ) scores participated in this study (Table 1). Participants with full-scale IQ scores below 70 were not included in the study. ASD participants were recruited from Kyushu University Hospital, and a local specialized psychiatric clinic. Diagnoses of ASD were confirmed, according to DSM-IV-TR criteria [31], by a clinical research team that included an experienced psychiatrist. Diagnostic agreement among the team was obtained for all participants. TD adults were recruited through public advertisements, and were interviewed to confirm the absence of any developmental or neuropsychiatric history, and/or medical conditions. All participants were right-handed, and had normal or corrected-to-normal visual acuity (> 1.0), evaluated using the Landolt ring [32]. No participants exhibited any color deficits, as determined by Ishihara color plates [33]. Written informed consent was obtained from each participant after the nature of the experiment had been fully explained. Experimental procedures were approved by the Ethics Committee of the Graduate School of Medical Sciences, Kyushu University.

**Table 1:** Demographic characteristics of ASD and TD adults.

Groups	Age (y)	Female/Male	FIQ	VIQ	PIQ
ASD (n = 14)	29.9 ± 7.1	5/9	104.9 ± 12.6	109.9 ± 14.9	97.3 ± 11.4
TD (n = 14)	28.6 ± 10.0	5/9	113.1 ± 9.4	115.0 ± 12.8	104.3 ± 12.0

Data are expressed as mean ± SD.

Abbreviations: *ASD*, autism spectrum disorder; *TD*, typically developing; *FIQ*, full scale intelligence quotient; *VIQ*, verbal intelligence quotient; *PIQ*, performance intelligence quotient

### Visual stimuli

**Fig 1:**
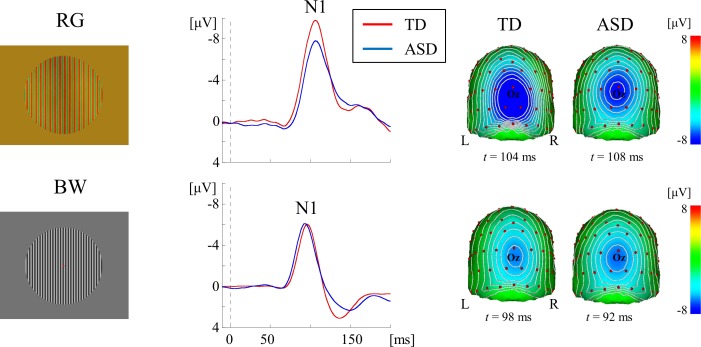
VEPs for RG and BW stimuli at the occipital ROI in each group. Grand-averaged waveforms show that N1 was a major component in both stimuli. Scalp topography shows N1 localized to the occipital area. *VEPs*, visual evoked potentials; *RG*, red/green; *BW*, black/white; *ROI*, region of interest.

**Fig 2:**
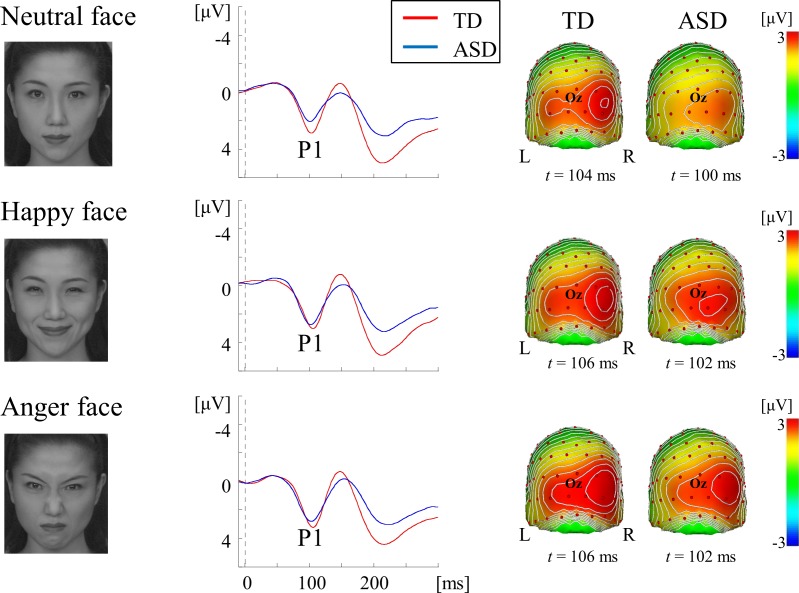
VEPs for face stimuli at the right occipital ROI in each group. Grand-averaged waveforms illustrate that P1 was evoked as a major component for all facial expressions. Scalp topography shows that P1 was distributed over the bilateral occipital area with right dominance.

**Fig 3:**
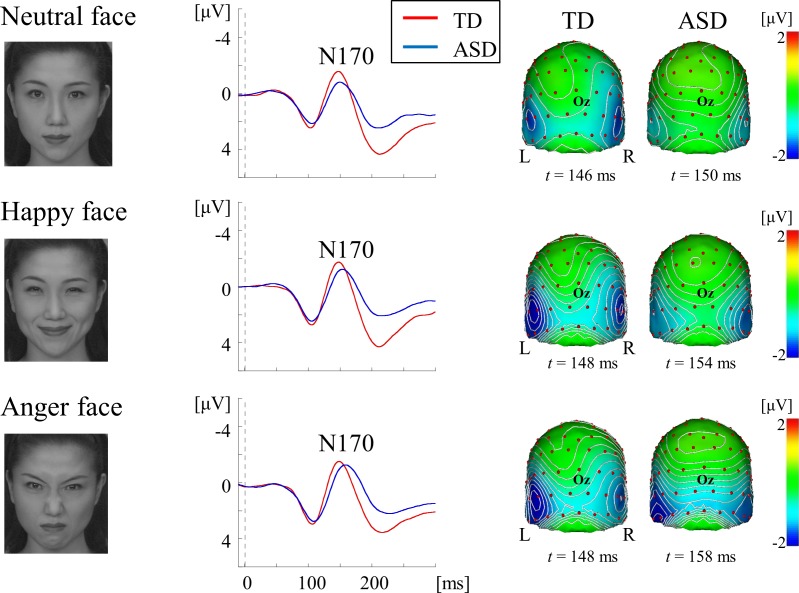
VEPs for face stimuli at the right occipitotemporal ROI in each group. Grand-averaged waveforms illustrate that N170 was evoked as a major component for all facial expressions. Scalp topography shows that N170 was distributed over the bilateral occipitotemporal area with right dominance.

We used three types of visual stimuli to systematically evaluate the function of the ventral stream. The combined use of isoluminant RG patterns with medium SF (2.0 cycles/degree, cpd), and high-contrast BW gratings with HSF (5.3 cpd) can selectively stimulate color and form pathways at the V1 level (Fig 1) [15]. Face stimuli preferentially activate the form pathway at both the lower (V1) and higher (V4) levels [16–19] (Figs 2 and 3).

#### RG stimulus

The isoluminant RG stimulus (10 × 10 degrees) was surrounded by a homogeneous background containing a mixture of red and green (thus appearing yellow) (Fig 1). Luminance for the stimulus and background was set to 21 cd/m^2^, and the SF was set to 2.0 cpd [15,20,22]. Before the experiment, subjects viewed a 15-Hz alternating red/green pattern stimulus to establish psychophysical isoluminance, and relative luminance was adjusted to minimize the perception of flicker [34].

#### BW stimulus

The BW stimulus (10 × 10 degrees) was surrounded by a homogeneous background containing a mixture of black and white (thus appearing gray) (Fig 1). The luminances for black and white were 0.5 and 41.5 cd/m^2^, respectively. The mean luminance of the achromatic gratings and the homogenous background was 21 cd/m^2^, and the contrast level was 98.8%. SF was set to 5.3 cpd [15].

#### Face stimuli

Individuals with ASD have greater difficulty recognizing facial emotions than TD individuals [2,35]. A recent meta-analysis showed that the sensitivity of the N170 component to facial expression is controversial [36] because early posterior negativity possibly confounds N170 [37,38]. Therefore, we adopted happy, angry, and emotionally neutral face stimuli (Figs 2 and 3).

Face stimuli (7.5 × 9.0 degrees; four females × three facial expressions and four males × three facial expressions) were 256-level grayscale photographs obtained from a commercially available database (the ATR face database DV99; ATR-Promotions, Inc., Kyoto, Japan) (Figs 2 and 3). Mean luminance and contrast were controlled by normalizing the mean and standard deviation (SD) of all stimuli (luminance, 70 ± 20 cd/m^2^).

### VEP paradigms

Visual stimuli were generated by ViSaGe (Cambridge Research Systems, Cambridge, UK) and were displayed on a gamma-corrected color monitor with a frame rate of 100 Hz (Electron22blue IV, LaCie, Tokyo, Japan). The distance between the monitor and participants was 114 cm. Each stimulus type was presented in a separate experiment comprising multiple consecutive sessions, with the order of the three experiments counterbalanced across participants. The total VEP recording time was approximately 20 min. No participants complained of fatigue during the experiments.

#### RG-VEPs

A trial began with presentation of the RG pattern (200 ms), followed by the homogeneous background (1000 ms), a cartoon character (1000 ms), and the homogeneous background (1000 ms) again (total trial length: 3200 ms). Cartoon characters (four boys, four girls, and two animals) were presented as the behavioral tasks (see Behavioral data). Participants completed 120 trials (four sessions × 30 trials/session) over the course of 5 min. The RG stimulus elicits the N1 component in the occipital region [15,20,22].

#### BW-VEPs

A trial began with presentation of the BW pattern (200 ms), followed by the homogeneous background (1000 ms), a cartoon character (1000 ms), and the homogeneous background (1000 ms) again (total trial length: 3200 ms). Cartoon characters (four boys, four girls, and two animals) were presented as the behavioral tasks (see Behavioral data), but were entirely different from those used in the RG-VEPs to avoid habituation to the cartoon characters. Participants completed 120 trials (four sessions × 30 trials/session) over the course of 5 min. The BW stimulus elicits the N1 component in the occipital region [15].

#### Face-VEPs

A trial comprised a randomly presented face stimulus on a gray background (300 ms), followed by presentations of a central fixation point (1000 ms) (total trial length: 1300 ms) [18,20]. Participants completed 525 trials (seven sessions × 75 trials/session; 175 trials in each stimulus) over the course of 12 min. Face stimuli elicit the right-lateralized occipital P1 and the occipitotemporal N170 as major components [18,20]. The P1 and N170 components originate from the V1 and V4, respectively [16–19,21].

### VEP recordings

VEPs were recorded using NetAmps 200, a high-density, 128-channel electroencephalographic (EEG) system (Electrical Geodesics Inc., Eugene, OR, USA). Amplified analog voltages were hardware band-pass-filtered at 0.1–200 Hz. The impedances of all channels were maintained below 50 kΩ [39]. EEG data were collected using the vertex (Cz) electrode as a reference. Participants sat on a chair in front of a monitor in a dark room, and fixated on a spot presented in the center of the monitor. They were instructed to remain relaxed and as motionless as possible, while fixating on the center of the screen with both eyes. Their arousal levels were carefully monitored visually by an observer within the room, and by EEG. If participants became drowsy, they were alerted and given a brief rest.

### Analysis of VEPs

Epochs that contained blinks, horizontal, or nonblank eye movements, or A/D saturation were rejected in offline analysis when constructing averaged waveforms. Electrodes surrounding the eyes were used to identify blinking and eye-movement artifacts. They were then re-referenced to the average reference offline. The analog data, hardware band-pass-filtered at 0.1–200 Hz, were digitized at a sampling rate of 500 Hz/channel, and filtered using a 1–30-Hz band-pass filter before averaging for all visual stimuli. Accepted samples were then averaged using EMSE Suite software (Cortech Solutions, Inc., USA). The number of accepted sweeps per condition across subjects was at least 80% of all samples.

**Fig 4:**
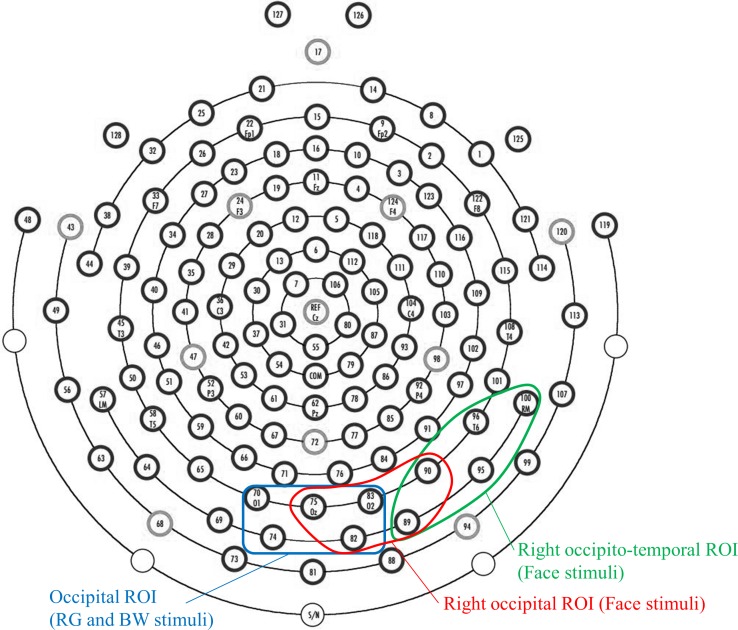
ROIs for each VEP measurement. We defined three ROIs: occipital ROI for N1 in response to RG and BW stimuli, and right occipital ROI for P1 and right occipitotemporal ROI for N170 in response to face stimuli.

We determined a region of interest (ROI) based on the topographic distribution of each VEP component (Fig 4). For RG- and BW-VEPs, five occipital electrodes were selected for the ROI of N1. For face-VEPs, the ROI of P1 was defined as five right occipital electrodes, while the ROI of N170 was defined as five right occipitotemporal electrodes. At least 100 samples in 400-ms epochs (from −100 to 300 ms) were averaged in RG- and BW-VEPs. For face-VEPs, at least 140 samples in 800-ms epochs (from −100 to 700 ms) were averaged.

Each VEP component was visually identified at a certain time window based on our earlier VEP studies [15,18,20,22], and post-hoc visual inspection of the VEP data. The N1 components for RG and BW were identified as the maximum negativity at the occipital region within a 80–160 ms latency window. P1 and N170 for faces were identified as the maximum positivity at the right occipital area within 70–140 ms, and maximum negativity at right occipitotemporal areas within 110–200 ms, respectively. Peak latencies were measured from stimulus onset, and peak amplitudes were measured from the pre-stimulus baseline to the peak of each stimulation in each subject.

Further, to examine cortico-cortical efficiency of visual information flow between V1 and V4, we calculated the difference in latency between P1 and N170 for face stimuli (N170 minus P1 [ms]), or between N1 for BW and N170 for face stimuli (N170 minus N1 [ms]) [17]. We also calculated the amplitude ratio (N170/P1 ratio [μV] and N170/N1 ratio [μV]) for V4/V1 imbalance. A photograph of a face is decomposed into several SF components [40]. In the wide ranging SF, the elemental HSF information of face configuration is comparable to that of the BW stimulus, which is mainly processed in the form pathway at V1 [15,41]. Here, we define the latency difference (“N170 minus N1 or P1”) and amplitude ratio (“N170/N1 or “N170/P1”) as the measures to evaluate the information flow from V1 to V4 within the form pathway. A larger latency difference or smaller amplitude ratio suggests deterioration of the form pathway. Conversely, the opposite pattern indicates facilitation of cortico-cortical form processing between V1 and V4 [17].

### Source analysis

Source localization of each VEP component was performed by standardized low resolution brain electromagnetic tomography (sLORETA [42,43]) using EMSE Suite software. In each VEP experiment, the current density was estimated at around peaks of each VEP component (time window: 20 ms; centered at the peak) of the grand-averaged waveforms in each group.

### Behavioral data

To maintain levels of arousal and attention, behavioral tasks were performed during VEP recordings. Participants were instructed to try to remember what cartoon characters were presented (RG and BW-VEPs), and what the facial expressions were (face-VEPs). After each VEP experiment condition, participants filled out a multiple-choice test (selecting one or more answers). This task was the same as in our earlier study [20].

### Statistical analyses

With regard to RG and BW-VEPs, we performed a two-way repeated-measures analysis of variance (ANOVA) to determine the effect of participant group (ASD or TD) and stimulus type (RG or BW) on the major component (N1). For analysis of face-VEPs, a two-way repeated-measures ANOVA was performed to determine the effect of participant group (ASD or TD) and stimulus type (neutral, happy, or angry face) on the major components (P1 and N170). We also performed a two-way repeated-measures ANOVA to determine the effect of participant group (ASD or TD) and stimulus type (neutral, happy, or angry face) on cortico-cortical latency differences (N170 minus P1 for faces, and N170 for faces minus N1 for BW), and amplitude differences (N170/P1 ratio and N170/N1 ratio). Bonferroni correction was applied to paired comparisons. A value of *p* < .05 was considered statistically significant. All statistical analyses were performed using SPSS 17.0 (SPSS Inc., Chicago, IL, USA). Raw data are provided as S1 Dataset.

## Results

### Neuropsychological and behavioral tests

There was no significant difference in IQ scores between ASD and TD participants (Table 1). With regard to behavior for RG- and BW-VEPs, after the VEP sessions, all participants correctly selected the cartoon characters that appeared during VEP recordings. Regarding face-VEPs, all participants also correctly identified facial expressions that appeared during VEP recordings.

### VEPs for RG and BW stimuli

**Fig 5:**
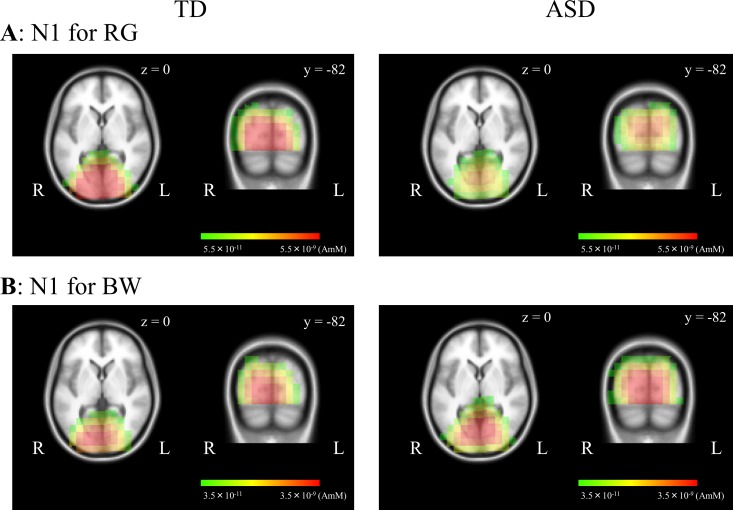
Source analysis for VEPs with RG and BW stimuli in each group. sLORETA activities were mapped onto the MNI space. N1 components for RG (A) and BW (B) stimuli were located at around occipital areas including V1 in both groups. *sLORETA*, standardized low resolution brain electromagnetic tomography; *MNI*, Montreal Neurological Institute.

In both stimuli, a negative component at approximately 90–120 ms (N1) was elicited as a major component in both groups (Fig 1). This component was located at the occipital area (maximally around channel 75 [Oz]), with no obvious difference in the distribution between the two groups in both stimuli (Fig 1). sLORETA estimation revealed activities that occured at around occipital areas including V1 in both RG (Fig 5A) and BW (Fig 5B) stimuli. In both stimuli, there was no obvious difference in the source localization between the two groups (Fig 5).

Regarding mean N1 latency, a significant main effect of stimulus type (F_(1,26)_ = 44.05, *p* < 0.001, partial η^2^ = 0.629) and interaction effect between stimulus type and participant group (F_(1,26)_ = 21.62, *p* < 0.001, partial η^2^ = 0.454) were observed, but the main effect of participant group was not significant. The mean N1 latency for RG stimulus was significantly prolonged in ASD adults compared with TD adults (*p* = 0.013). In contrast, the mean N1 latency for BW stimulus was significantly shorter in ASD adults compared with TD adults (*p* < 0.001) (Table 2A). In addition, the mean N1 latency for BW stimulus was significantly shorter compared with RG stimulus in ASD (*p* < 0.001), but not TD adults (Table 2A). With regard to mean N1 amplitude, there were no significant main or interaction effects (Table 2A).

**Table 2:** VEPs for RG, BW, and face stimuli in ASD and TD adults.

(A) N1 for RG and BW stimuli (occipital ROI)				
Stimuli	Amplitude (μV)		Latency (ms)	
	ASD	TD	ASD	TD
RG	9.5 ± 6.6	9.9 ± 6.0	115.5 ± 14.1^#^^,^^***^	103.5 ± 9.3
BW	6.6 ± 4.3	5.2 ± 3.4	90.7 ± 5.7^##^	99.1 ± 3.8
(B) P1 for face stimuli (right occipital ROI)				
Stimuli	Amplitude (μV)		Latency (ms)	
	ASD	TD	ASD	TD
Neutral	3.0 ± 1.3	3.2 ± 2.8	101.8 ± 12.2	109.9 ± 10.0
Happy	3.4 ± 1.1	3.5 ± 2.9	103.2 ± 11.7	110.4 ± 8.8
Anger	3.6 ± 1.4	3.6 ± 2.9	102.5 ± 10.5	111.4 ± 9.1
(C) N170 for face stimuli (right occipitotemporal ROI)				
Stimuli	Amplitude (μV)		Latency (ms)	
	ASD	TD	ASD	TD
Neutral	1.8 ± 1.4	2.1 ± 2.5	154.1 ± 14.7	144.2 ± 11.3
Happy	2.0 ± 1.5	2.2 ± 2.5	154.9 ± 12.5	146.1 ± 10.6
Anger	1.7 ± 1.9	2.0 ± 2.5	157.0 ± 15.2	146.8 ± 11.8

Data are expressed as mean ± SD.

ASD vs. TD

^##^, *p* < 0.001,

^#^, *p* < 0.05;

RG vs. BW

***, *p* < 0.001

Abbreviations: *VEPs*, visual evoked potentials; *ROI*, region of interest; *RG*, red/green; *BW*, black/white

### VEPs for face stimuli

**Fig 6:**
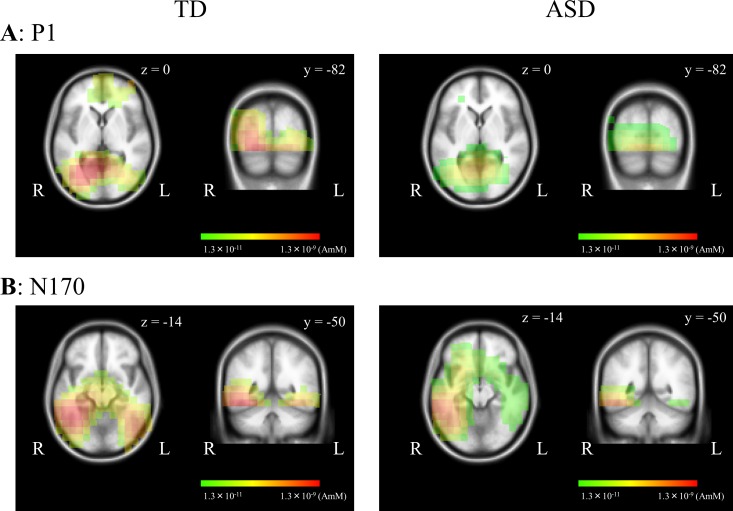
Source analysis for VEPs with face stimuli in each group. sLORETA activities were mapped onto the MNI space. P1 (A) and N170 (B) components for neutral face stimuli were located at around occipital areas including V1 and occipitotemporal areas including V4, respectively, in both groups.

For both groups, a positive component at approximately 100–110 ms (P1), and a negative component at approximately 140–160 ms (N170) were elicited as major components (Figs 2 and 3). The P1 component was distributed over occipital areas with right-hemisphere dominance (maximally around channel 83 [O2], 82 and 89) (Fig 2). The N170 component was distributed over bilateral occipitotemporal areas with right-hemisphere dominance (maximally around channels 95 and 100) (Fig 3). Fig 6 shows the results of sLORETA estimation in P1 and N170 components for the neutral face stimulus. The source of P1 was estimated in the occipital areas including V1 (Fig 6A), while that of N170 was located at around occipitotemporal areas including V4 (Fig 6B). No obvious difference was found in source localization between the two groups (Fig 6). sLORETA maps of happy and anger face stimuli revealed similar findings to that of the neutral face stimulus (data not shown).

With regard to mean P1 latency, a significant main effect of participant group (F_(1,26)_ = 4.292, *p* = 0.048, partial η^2^ = 0.142) was found. Regardless of stimulus types (facial expression), mean P1 latency was significantly shorter in ASD adults compared with TD adults (*p* = 0.048) (Table 2B). There was no significant main effect of stimulus type or interaction effect between stimulus type and participant group. No significant main or interaction effects were found for mean P1 amplitude (Table 2B).

For mean N170 latency, the main effect of participant group was significant (F_(1,26)_ = 4.296, *p* = 0.048, partial η^2^ = 0.142). Regardless of stimulus type (facial expression), mean N170 latency was significantly longer in ASD adults compared with TD adults (*p* = 0.048) (Table 2C). No significant main effect of stimulus type or interaction effect between stimulus type and participant group was found. Regarding mean N170 amplitudes, there were no significant main or interaction effects (Table 2C).

### Cortico-cortical information flow between V1 and V4

There was a significant main effect of difference in latency between P1 and N170 (N170 minus P1) for faces (F_(1,26)_ = 35.662, *p* < 0.001, partial η^2^ = 0.578). Regardless of stimulus type (facial expression), this difference in latency was significantly larger in ASD adults compared with TD adults (*p* < 0.001) (Table 3A). No significant main effect of stimulus type or interaction effect between stimulus type and participant group was observed. Regarding the amplitude ratio between P1 and N170 (N170/P1), there were no significant main or interaction effects (Table 3A).

**Table 3:** Comparison between lower (V1) and higher (V4) cortical responses in ASD and TD adults.

(A) P1 and N170 for face stimuli				
Stimuli	Amplitude ratios (N170/P1) (μV)		Latency difference (N170 minus P1) (ms)	
	ASD	TD	ASD	TD
Neutral	0.8 ± 1.3	1.2 ± 1.4	52.3 ± 6.7	34.4 ± 8.1
Happy	0.5 ± 0.3	1.2 ± 1.6	51.7 ± 7.1	35.7 ± 9.0
Anger	0.7 ± 1.0	1.0 ± 1.1	54.5 ± 10.9	35.4 ± 9.7
(B) N1 for BW stimulus and N170 for face stimuli				
Stimuli	Amplitude ratios (N170/N1) (μV)		Latency difference (N170 minus N1) (ms)	
	ASD	TD	ASD	TD
Neutral	0.5 ± 0.7	0.4 ± 0.8	63.4 ± 12.4	45.1 ± 11.2
Happy	0.5 ± 0.7	0.4 ± 0.7	64.2 ± 10.0	47.0 ± 10.5
Anger	0.5 ± 0.7	0.4 ± 0.7	66.3 ± 12.8	47.7 ± 11.5

Data are expressed as mean ± SD.

Similarly, the main effect of difference in latency between N1 for BW and N170 for faces (N170 minus N1) was significant (F_(1,26)_ = 19.267, *p* < 0.001, partial η^2^ = 0.426). This difference in latency was significantly larger in ASD adults compared with TD adults regardless of stimulus type (facial expression) (*p* < 0.001) (Table 3B). However, the main effect of stimulus type and interaction effect between stimulus type and participant group were not significant. In addition, there were no significant main or interaction effects for amplitude ratios between N1 and N170 (N170/N1) (Table 3B).

## Discussion

To our knowledge, this is the first electrophysiological study to systematically evaluate the function of the color and form pathways within the ventral stream in ASD. All participants correctly performed the behavioral tasks. Our electrophysiological results were, therefore, considered to be predominately unaffected by levels of arousal or attention. Specifically, we found that compared with TD controls, ASD adults exhibited longer N1 latency for RG stimuli, shorter N1 latency for BW, and shorter P1 latency, but prolonged N170 latency for face stimuli. Moreover, we found a larger difference in latency between P1 and N170 for faces, or between N1 for BW and N170 for faces. These findings indicate that distinct functional alterations are present in the ventral stream from V1 to V4 in ASD adults.

### Enhanced form pathway function at the V1 level

In BW-VEPs, significantly shorter latency of N1 (V1 origin) was found in ASD adults compared with TD adults, indicating enhanced form pathway function at V1 in ASD. Recently, Kéïta et al. [44] demonstrated that ASD participants were more sensitive to luminance-defined HSF gratings (8 cpd) compared with lower SF gratings (0.5, 1, 2, and 4 cpd). A group difference in peak distribution was also observed, as 35% of ASD participants manifested peak sensitivity for luminance-defined gratings of 4 cpd, compared with only 7% for the control group. Using VEPs, Vlamings et al. [45] reported enhanced P1 activity to HSF compared with LSF grating in children with ASD compared with controls. These behavioral and VEP studies suggest a processing bias for HSF information at the V1 level in ASD. Because the BW stimulus used in the current study comprised HSF information (5.3 cpd), shorter N1 latency in our study is considered to reflect enhanced fine-form (HSF) processing at V1 in ASD.

### Impaired color pathway function at the V1 level

In RG-VEPs, ASD adults exhibited prolonged latency of N1 (V1 origin) compared with TD adults, indicating that function of the color pathway at V1 is impaired in ASD adults. In our previous VEP study, we found significantly prolonged N1 latency for chromatic (RG) gratings (the same visual stimulus used in this study) in ASD adults compared with TD adults [22]. Thus, our results showed a robust reproducibility even though ASD participants in the current study were completely different from those in the study of Fujita et al. [22]. Further, our VEP studies support psychological findings that atypical color perception and cognition exist in high-functioning ASD [2,46,47].

In V1, the color pathway (blob region) is characterized by a rich concentration of mitochondrial cytochrome oxidase (CO), whereas the form pathway (interblob region) has lower CO concentration. CO is the last enzyme in the mitochondrial respiratory chain engaged in oxidative metabolism and energy production [48–50], and is closely coupled to neuronal functional activity [48]. Interestingly, mitochondrial dysfunction in the brains of ASD individuals has been recently reported in neuroimaging and postmortem studies [51,52]. Thus, deficits in the color pathway in ASD may reflect mitochondrial dysfunction in the blob region of V1. Subsequently, this impairment may induce compensatory enhancement of form pathway function in ASD, because the color pathway anatomically interacts with the form pathway [53].

### Enhanced V1 function and impaired global integration at the V4 level during face perception in ASD

For face-VEPs, ASD adults exhibited significantly shorter latency of P1 (V1 origin) compared with TD adults. This result suggests enhanced low-level physiological processing of faces at V1 in ASD, because P1 reflects processing of low-level physiological properties including contrast, luminance, and SF of visual stimuli [41]. A recent meta-analysis of neuroimaging studies demonstrated that early visual areas (striate and extrastriate areas) were activated to a greater degree in individuals with ASD compared with those without ASD during face processing tasks [54]. This finding supports our result of enhanced V1 function in ASD adults.

Faces are much more complex stimuli (broadband SF stimuli). Thus, they are processed through integration of co-occurrent fine HSF and coarse LSF information arising from the HSF (form) pathway and LSF (dorsal and subcortical) pathway, respectively [55]. Earlier neuropsychological studies reported that ASD individuals might be more biased in favor of HSF than LSF in faces [56,57]. In a VEP study, the emotional effect at P1 was significant for HSF, but not LSF in an ASD group [45]. Accordingly, the present result of enhanced V1 function may reflect enhanced fine-form perception related to local HSF processing of faces at V1 in ASD adults. This finding is consistent with that of BW-VEPs.

Interestingly, ASD adults exhibited longer latency of N170 (V4 origin), and a larger latency difference between P1 and N170 compared with TD adults during face perception. Similarly, ASD adults showed a larger latency difference between N1 for BW gratings and N170 for face stimuli compared with TD adults. These findings indicate impairment of higher-level function of the form pathway in ASD. Several previous neuroimaging studies demonstrated impairment of the fusiform gyrus in ASD [58–60], but comparable functional properties of the fusiform gyrus between ASD and controls were also reported [61–64]. Another neuroimaging study also reported decreased functional connectivity of the face processing network, including the fusiform gyrus and amygdala, when ASD participants processed face stimuli, suggesting dysfunction at the network level, rather than at each constituent node [65]. Together, the present findings suggest that ASD adults have dysfunction of V4 *per se*, and/or the cortico-cortical network between V1 and V4 within the form pathway.

Consistent with the present study, some VEP studies reported delayed N170 latencies for face stimuli in ASD compared with controls [25–27], however, other studies found no latency difference for N170 [28–30]. This was probably due to the fact that the previous VEP studies used different stimuli and tasks in different age groups and different levels of ASD severity. However, in a recent systematic review [66], two of three VEP studies in ASD adults showed delayed N170 latencies in facial emotion discrimination or identification tasks [26,27]. Our study also used the facial expression memory task. Thus, VEPs with facial emotion-related tasks may easily cause prolonged N170 latency in ASD.

Using functional magnetic resonance imaging with hybrid face stimuli, Corradi-Dell’Acqua et al. [55] investigated the independent contribution of HSF and LSF visual input to brain regions critical for face processing in ASD. ASD individuals showed reduced fusiform response to HSF, but intact response to LSF. The authors concluded that ASD might be characterized by difficulty in integrating multiple local information causing global processing difficulties related to HSF information. Therefore, the present results of prolonged N170 latency, and larger differences in latency between P1 and N170 or between N1 and N170 may reflect deficit in the integration of multiple local HSF information of faces into gestalt information at the V4 level in ASD. In sum, the present study demonstrated enhanced fine-form processing at V1 and impaired gestalt face processing at V4 in individuals with ASD.

Overall, the most valuable point of the present study was the implementation of simultaneous functional evaluation to each pathway within the ventral stream of individuals with ASD. Our novel findings are rather complicated but suggest functional alteration of the ventral pathway in ASD adults: Coexistence of enhanced function of the form pathway with an impaired color pathway at V1 and an impaired form pathway at V4. Therefore, the present study provides new insight into the neural basis of atypical visual and social processing in ASD.

### Future directions

Some behavioral studies have suggested that facial color is important for face perception [67,68]. Facial color provides useful clues to estimating the mental or physical condition of another person [69]. Further, recent VEP studies demonstrated that P1 and N170 components are modulated by facial color in healthy adults [68,69]. These VEP studies proposed that P1 modulation reflected low-level physical characteristics processing, whereas N170 modulation was caused by the processing of facial color information [68,69]. Because the present study used grayscale photographs, further VEP studies to compare grayscale with colored face stimuli will be needed to more fully elucidate the altered functional interaction between color and form pathways in individuals with ASD.

## Conclusions

Individuals with ASD show enhanced fine-form (local HSF) processing with impaired color processing at the V1 level. In contrast, they exhibit impairment of gestalt face processing due to deficit in integrating multiple local HSF information of faces at the V4 level. These functional alterations of the ventral stream may be an important factor in atypical social processing in ASD.

## Supporting Information

S1 Dataset The data for VEP amplitudes and latencies. (XLSX)
